# ISENICS: a model for identifying senescent immune cells and samples and characterization of their roles in tumor microenvironment

**DOI:** 10.1093/bib/bbaf469

**Published:** 2025-09-11

**Authors:** Miaomiao Tian, Hao Cui, Xinyu Wang, Huading Hu, Longlong Dong, Song Xiao, Changfan Qu, Peng Wang, Hui Zhi, Shangwei Ning, Yue Gao

**Affiliations:** College of Bioinformatics Science and Technology, Harbin Medical University, No. 157 Baojian Road, Nangang District, Harbin City, Heilongjiang Province, 150081, China; The Second Affiliated Hospital of Harbin Medical University, No. 148 Baojian Road, Nangang District, Harbin City, Heilongjiang Province, 150081, China; College of Bioinformatics Science and Technology, Harbin Medical University, No. 157 Baojian Road, Nangang District, Harbin City, Heilongjiang Province, 150081, China; College of Bioinformatics Science and Technology, Harbin Medical University, No. 157 Baojian Road, Nangang District, Harbin City, Heilongjiang Province, 150081, China; College of Bioinformatics Science and Technology, Harbin Medical University, No. 157 Baojian Road, Nangang District, Harbin City, Heilongjiang Province, 150081, China; The Second Affiliated Hospital of Harbin Medical University, No. 148 Baojian Road, Nangang District, Harbin City, Heilongjiang Province, 150081, China; College of Bioinformatics Science and Technology, Harbin Medical University, No. 157 Baojian Road, Nangang District, Harbin City, Heilongjiang Province, 150081, China; College of Bioinformatics Science and Technology, Harbin Medical University, No. 157 Baojian Road, Nangang District, Harbin City, Heilongjiang Province, 150081, China; College of Bioinformatics Science and Technology, Harbin Medical University, No. 157 Baojian Road, Nangang District, Harbin City, Heilongjiang Province, 150081, China; College of Bioinformatics Science and Technology, Harbin Medical University, No. 157 Baojian Road, Nangang District, Harbin City, Heilongjiang Province, 150081, China; College of Bioinformatics Science and Technology, Harbin Medical University, No. 157 Baojian Road, Nangang District, Harbin City, Heilongjiang Province, 150081, China

**Keywords:** immune cellular senescence, tumor immune microenvironment, immune response, prognosis

## Abstract

Senescent immune cells secrete varied inflammatory factors that weaken the systemic anti-tumor ability and promote the proliferation and metastasis of tumor cells. Tumor cells could also accelerate the immune cellular senescence through diverse mechanisms. However, there has been a lack of indicators to quantify the senescence levels of different immune cell types. A model for Identifying Senescent Immune Cells and Samples was developed to explore the role of senescent immune cells in the tumor immune microenvironment (TIME). By integrating bulk and single-cell RNA-seq data, we constructed immune cell gene expression profiles for 23 cancer types using a deconvolution algorithm. By calculating the cellular senescence scores, we found that tumor samples exhibited higher senescence levels than normal samples. Monocytes/macrophages were prone to co-senescence with other cell subtypes. Differentially expressed genes in the high- and low-immune cellular senescence scores groups were enriched in the senescence pathway. Patients with higher levels of immunosenescence were associated with better prognosis. At the single-cell level, the number and strength of cell-to-cell interactions increased following immune cellular senescence in most cancers. Samples with senescent immune cells exhibited poorer immunotherapy response. Our study advances our understanding of senescent immune cells in the TIME, provides insights into cancer-specific relationships between immune cellular senescence and immune characteristics, and offers a model for identifying these senescent immune cells.

## Introduction

Senescent immune cells, characterized by irreversible cell cycle arrest, are one of the most pathogenic subsets of senescent cells. These cells are often characterized by the Senescence-Associated Secretory Phenotype (SASP), macromolecular damage, and metabolic dysfunction [1]. Senescent immune cells exhibit an inability to re-enter the cell cycle despite mitotic stimuli, an enhanced secretory phenotype, and resistance to cell death [2, 3]. By accelerating solid organ senescence, they contribute to systemic aging [4]. Immunosenescence is characterized by altered T-cell ratios, impaired calcium-mediated signaling, and thymic atrophy. These changes led to a reduction in the number of immune cells and increased expression of CDKN2A (p16), CDKN1A (p21), and SASP [5, 6], thereby accelerating the accumulation of senescent cells and promoting tumorigenesis [7, 8]. This highlights senescent immune cells as a key driver of cancer progression. Investigating senescent immune cells in different cancer types may provide breakthrough insights into how senescent immune cells contribute to tumor progression and treatment resistance [9]. Regulating immune senescence and enhancing immune response are novel strategies for preventing and treating malignant tumors [10].

Previous research has demonstrated that senescent cells typically overexpress cell cycle regulators like p16 and p21, which mediate cell cycle arrest [11]. However, these markers lack specificity for senescent immune cells, as not all p16-positive immune cells are senescent, and some senescent immune cells may not express p16 or p21 [12]. The majority of research has focused on the tissue-level senescence, with limited exploration of cell-type-specific senescence across cancers [13]. As a result, the role of senescent immune cells in the tumor immune microenvironment (TIME) and their impact on tumor recurrence remains poorly understood.

While bulk RNA-seq offers cost-effectiveness, scalability, and clinical utility, it fails to resolve cell heterogeneity. Conversely, single-cell RNA-seq provides high resolution but struggles to capture large or fragile senescent immune cells, requires fresh/frozen specimens rather than formalin-fixed tissues, and imposes technical challenges for rare cell populations [14, 15]. To address these limitations, we apply deconvolution to The Cancer Genome Atlas (TCGA) bulk RNA-seq datasets, leveraging their clinical richness to reconstruct cell-type-specific gene expression [16]. Unlike traditional methods based on linear regression or non-negative matrix factorization, the Tissue-Adaptive autoEncoder (TAPE) employs unsupervised learning to capture tissue-specific expression patterns, integrates supervised signals from marker genes, and generates pseudo-cell bulk matrices [16]. In its default “overall” mode, TAPE predicts cell proportions based on input single-cell gene expression profiles from the same tissue. It was primarily used to predict cell proportions for the whole, but it does not generate a signature matrix individually for each sample. The “high-precision” model allowed for prediction of the cell-type-specific gene expression profiles for each sample.

Here, we present ISENICS (Identification of Senescent Immune Cells and Samples), an integrated framework combining Gene Set Variation Analysis (GSVA) and TAPE-based deconvolution to (i) infer gene expression profiles of immune cells from a large number of tissue samples, (ii) identify senescent immune cells and (iii) categorize the patients into High and Low senescent TIME (HSTIME and LSTIME) groups. Gene sets from published methods were used to compare and select during the calculation of the immune cellular senescence scores (ICSS). Using ISENICS, we found that tissue type may play a critical role in influencing the senescence levels in cancers, with tumor growth potentially representing a mechanism to evade cell senescence. Furthermore, HSTIME patients had a better survival trend than LSTIME patients in KIRC, LAML, SKCM, THCA, and integrated datasets from 23 types of cancer. The number and strength of cell-to-cell interactions increased after immune cellular senescence in pan-cancer. We also focus on the interactions between monocytes/macrophages (Mono/Macro) and other immune cells in the senescent TIME. Notably, senescent cell-containing samples exhibited inferior immunotherapy response, prompting us to develop a multimodal machine learning predictor for treatment outcome anticipation.

## Methods

### Data collection

Bulk RNA-seq transcriptional expression profiles and associated clinical data for 23 cancer types (14 382 samples, Supplementary Table 1) were obtained from two databases: TCGA (https://portal.gdc.cancer.gov) and the Genotype-Tissue Expression (GTEx, https://www.gtexportal.org/home/). Information from validation cohorts was downloaded from the Gene Expression Omnibus (GEO, https://www.ncbi.nlm.nih.gov/geo) database under the accession numbers GSE173377, GSE275256, GSE30240, and GSE97862.

Single-cell RNA-seq data downloaded from the Tumor Immune Single-cell Hub database (TISCH, http://tisch.comp-genomics.org/home/) include BLCA (GSE145281, 14 474 cells), BRCA (GSE114727, 19 676 cells), CESC (GSE168652, 22 998 cells), CHOL (GSE125449, 5762 cells), COAD (GSE146771, 10 468 cells), DLBC (GSE175510, 1412 cells), ESCA (GSE173950, 9544 cells), GBM (GSE163108, 8244 cells), HNSC (GSE103322, 5902 cells), KICH (GSE159115, 2850 cells), KIRC (GSE171306, 11 427 cells), LAML (GSE154109, 10 799 cells), LIHC (GSE140228, 7074 cells), LUAD (GSE127465, 31 179 cells), LUSC (GSE127465, 31 179 cells), OV (GSE147082, 9796 cells), PAAD (GSE111672, 6122 cells), PRAD (GSE137829, 25 436 cells), SKCM (GSE72056, 4645 cells), STAD (GSE167297, 22 464 cells), THCA (GSE148673, 19 407 cells), UCEC (GSE139555, 12 758 cells), and UVM (GSE138433, 12 682 cells). These datasets were utilized for deconvolution. Validation cohorts included BLCA (GSE149652, 15 538 cells), CHOL (GSE142784, 10 535 cells), ESCA (GSE154763, 7673 cells), GBM (GSE141982, 5263 cells), GBM (GSE84465, 3533 cells), LIHC (GSE125449, 3834 cells), LUAD (GSE149655, 1412 cells), LUAD (GSE153935, 3658 cells), LUAD (GSE146100, 10 996 cells), LUSC (GSE153935, 3658 cells), LUSC (GSE150660, 5407 cells), LUSC (GSE117570, 11 453 cells), OV (GSE130000, 11 215 cells), PAAD (GSE154763, 2853 cells), PAAD (GSE148673, 6196 cells), SKCM (GSE115978, 16 291 cells), THCA (GSE154763, 5312 cells), THCA (GSE163203, 1561 cells), and UCEC (GSE154763, 8808 cells). Immunotherapy cohorts, including GSE203115 (12 762 cells), GSE145281 (14 474 cells), and GSE207422 (92 330 cells), which contained a total of 17 responders and 20 nonresponders, and the senescence cohort GSE115301 (480 cells) were collected from the GEO database.

### Establishment of identifying senescent immune cells and samples model

**Figure 1:**
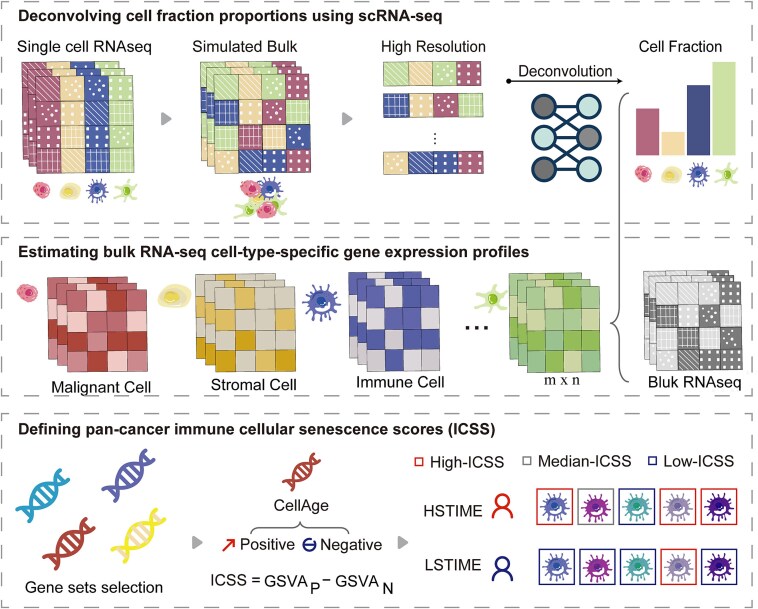
Integrative framework for pan-cancer cellular senescence analysis. Workflow for integrative analysis of the cellular senescence landscape across cancers using deconvolution.

We developed the ISENICS model, which includes three steps (Fig. 1). (i) **Deconvolving cell fraction proportions using scRNA-seq**: To maintain consistency between single-cell (TPM from TISCH) and bulk RNA-seq data, we used TCGA TPM expression profiles as input for TAPE. Bulk and single-cell gene expression profiles were imported into the scTAPE Python package (v1.1.2) with the “datatype” parameter set to “TPM”, the “mode” parameter set to “high-resolution”, and the “adaptive” parameter set to “True”. Using single-cell RNA-seq data, TAPE generated pseudo-bulk data to optimize its loss functions and learn parameters. After that, TAPE deconvolved the cell infiltration proportions across different cancers. (ii) **Estimating bulk RNA-seq cell-type-specific gene expression profiles**: The specific TPM expression profiles of cell subtypes were predicted for each bulk sample, with cell types matching those in the scRNA-seq data. On the one hand, for cancer types with multiple single-cell RNA-seq data collections, we performed multiple single-cell deconvolutions to generate expression profiles. The robustness of the TAPE was assessed by calculating the correlation significance *P-*values between these deconvolved expression profiles. On the other hand, 40 samples were randomly taken from each expression profile to generate a subset with 7600 samples, and we checked the expression of cell marker genes. Finally, we screened immune cell expression profiles for subsequent analysis, including those of B cells, CD4+ conventional T cells (CD4Tconv), CD8+ T cells (CD8T), exhausted CD8+ T cells (CD8Tex), dendritic cells (DC), mast cells, monocytes/macrophages (Mono/Macro), neutrophils, natural killer cells (NK), plasma cells, proliferating T cells (Tprolif), and regulatory T cells (Treg). (iii) **Defining pan-cancer ICSS**: We collected four previously published senescence-related gene sets: CellAge [17], SenMayo [18], siAge [19], and SenCID [20]. The CellAge and the siAge gene sets categorized genes into senescence-positive and senescence-negative gene sets. The ICSS for the CellAge and siAge gene sets were defined as the difference between the GSVA activity scores of their positive and negative gene sets, using the GSVA R package (v2.0.7). However, the SenMayo and SenCID gene sets do not have such positive or negative categories, so we defined ICSS for SenMayo and SenCID as the GSVA scores of their gene sets directly. We collected four immune cellular bulk RNA-seq datasets GSE173377, GSE275256, GSE30240, and GSE97862 (Supplementary Table 2). To select a relatively precise gene set for model construction, we respectively calculated the ICSS differences for each gene set in four immune cellular datasets. Ultimately, the CellAge gene set was found to be superior in recognizing senescent immune cells and was used to construct the ISENICS model.

In addition, we collected 91 genes of SASP from literature and calculated the correlation of ICSS with SASP gene expressions, as well as the proportion of positively correlated SASP genes among the expressed SASP genes. The first and third ICSS quartiles were used as thresholds for categorizing the samples into high-, medium-, and low-cellular senescence scores groups. The cellular senescence scores calculations were performed for GTEx normal samples, TCGA tumor samples, and TCGA tumor-adjacent samples to assess the variations across different tissue types. Immunohistochemistry (IHC) staining was downloaded from The Human Protein Atlas (https://www.proteinatlas.org/) to confirm protein-level senescence markers [21].

We calculated the ICSS for individual immune cells based on the deconvolved expression profiles. Senescent co-occurring cell types were identified through network analysis. In order to verify the accuracy of ICSS in the assessment of senescence, the ICSS of four datasets with senescent and nonsenescent samples was calculated separately, and the significance of differences was determined.

TCGA tumor patients were categorized into three subgroups: patients with more than half of the senescent immune cell subtypes (high-ICSS) were classified as the High Senescent Tumor Immune MicroEnvironment (HSTIME) group. Conversely, patients with more than half of the nonsenescent immune cell subtypes (low-ICSS) were classified as the Low Senescent Tumor Immune MicroEnvironment (LSTIME) group. Patients who did not meet the criteria for HSTIME or LSTIME were classified as the Median Senescent Tumor Immune MicroEnvironment (MSTIME) group. In conclusion, the ISENICS model enabled a thorough investigation of the complex relationship between senescent immune cells and TIME across 23 cancer types.

### Differential expression analysis between distinct groups using ISENICS

The medium-ICSS group was excluded due to low variance in ICSS distribution. Differentially expressed genes (DEGs) were identified in TCGA tumor gene expression profiles (HSTIME vs. LSTIME) and deconvolved immune cell-type-specific gene expression profiles (high-ICSS vs. low-ICSS) using the limma R package (v3.60.0). Significance thresholds were *P* < .01 and |FC| > 2 (bulk) or |FC| > 1.2 (immune cells). Finally, DEGs of immune cells between high- and low-ICSS groups were functionally enriched via Gene Ontology enrichment analysis.

To determine the correlation between DEGs between ICSS groups and immune response, we curated immune checkpoint (ICP) and immune-related genes from published literature. Fisher’s exact test was then used to calculate *P-*values and to confirm the significance of the overlaps among these gene sets.

### Pan-cancer analysis of ISENICS-classified samples

We implemented algorithms from the IOBR R package (v0.99.8) [22], including the Tumor Immune Estimation Resource (TIMER) [23], Estimation of Proportions of Immune and Cancer cells [24], Microenvironment Cell Populations-counter [25], Estimation of Stromal and Immune cells in Malignant Tumor tissues using Expression data (ESTIMATE) [26], and Quantification of the Tumor Immune contexture from human RNA-seq data [27]. Correlations between ICSS and immune infiltration scores assessed by IOBR were computed and visualized via heatmaps to link senescence levels with immune infiltration.

Differences in GSVA scores of immune-related gene sets and survival probability between HSTIME and LSTIME were compared. To investigate ISENICS-related survival, we constructed Cox proportional hazards regression models for overall survival (OS), with sample groups set as time-dependent covariates and cancer types set as factors. hazard ratios with 95% confidence intervals (CI) and *P-*values were visualized. Kaplan–Meier curves with log-rank tests quantified survival differences. Survival analyses were performed using the survival (v3.6.4) and survminer (v0.4.9) R packages. The Benjamini–Hochberg method was used to control the FDR, correcting the *P*-value.

### Comparative analysis of intercell communication between senescent and nonsenescent cells using single-cell RNA-seq

For single-cell RNA-seq data, we defined ICSS using AUCell scores from the irGSEA R package (v3.3.2). The sensitivity and specificity of the ISENICS were evaluated using the pROC R package (v1.18.5) in the GSE115301 dataset. Immune cells with the top 25% and bottom 25% ICSS from single-cell RNA-seq data were assigned to the high-ICSS and low-ICSS groups, respectively. The uniform manifold approximation and projection (UMAP) clustering was visualized using the Seurat R package (v4.4.0; Supplementary Fig. 1). Additionally, pseudotime analysis was conducted to track changes in ICSS over time, utilizing the monocle2 R package (v2.32.0; Supplementary Fig. 2).

The CellChat R package (v1.6.1) inferred immune cell communication networks between high- and low-ICSS groups. Subsequently, we compared the differences in both the number and strength of intercell communication between the two groups. We calculated the total number of cell communications, with a focus on Mono/Macro cells as the most abundant cell type. A separate visualization was generated to illustrate interplays between Mono/Macro cells and other immune cell types. Immune cells from the high- and low-ICSS groups across all cancer types were pooled together for further analysis. Batch effects were corrected using the harmony R package (v1.2.0). Differences in cell interactions were then calculated, and the overall information flow between ICSS groups was assessed for each signaling pathway. Additionally, upregulated and downregulated ligand-receptor pairs were visualized by ICSS group.

### Evaluating the predictive value of ISENICS for immunotherapy response

We analyzed immunotherapy datasets (GSE203115, GSE145281, GSE207422) with annotated response information. Genes detected in ≥5 cells and cells with ≥200 detected genes were retained. The first 30 principal components were extracted, and the resolution was set to 0.5. We annotated cell subtypes manually and calculated ICSS for each dataset. After removing the double cells and the environmental pollution effect, three datasets were integrated. The chi-square test was conducted to assess the significance of differences in immunotherapy response between ICSS groups in different immune cells.

DEGs between ICSS groups were identified as *P* ≤ .05 and |FC| > 2. DEGs with a *P* ≤ .05 in a single-factor Cox regression model were selected for further analysis using the Least Absolute Shrinkage and Selection Operator (LASSO) regression model to screen for genes predictive of immune response. The resulting genes were utilized to construct the immune response predictor. The importance of each CellAge gene among the resulting genes was assessed using the glm function in R.

Samples were split into training (70%) and testing (30%) cohorts. Our immune response predictor was constructed using some machine learning models from the Mime1 R package (v0.0.0.9): Naive Bayes, Random forest, K-nearest neighbors, AdaBoost classification tree, and Boosted logistic regression. The areas under the Curve (AUCs) for individual cohorts were calculated using the pROC R package (v1.18.5). We also calculated the AUCs for other clinical features to compare them with our classifier.

### Statistical analysis

Kruskal–Wallis tests compared ≥3 non-normally distributed groups. Spearman’s correlation was applied to evaluate the correlation coefficient. Significance thresholds: ^*^P ≤ .05, ^**^P ≤ .01, ^***^P ≤ .001, ^****^P ≤ .0001; ns: nonsignificant (*P* > .05). All statistical analyses were carried out using R.

## Results

### Computing cell-type-specific gene expression profiles across cancer types

**Figure 2:**
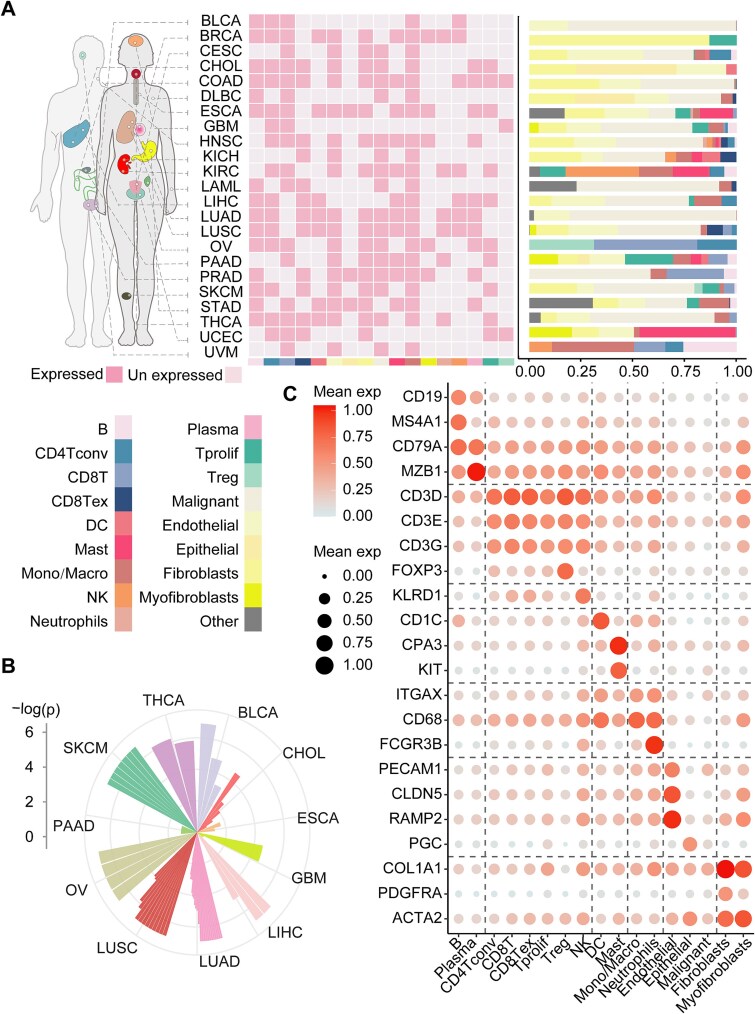
Deconvolution of cell-type-specific gene expression profiles was diverse. (A) The heatmap illustrates the cell subtypes (have more than two deconvolved matrices) and bar plot showing cell infiltration levels for each cancer type. (B) Polar plot illustrates significant *p*-values associated with deconvolved matrix pairs. (C) Dot plot illustrates marker genes expression levels of each cell type.

In the TISCH database, single cells for 23 cancer types were annotated into three major types: malignant cells, stromal cells, and immune cells. Cell type distribution varied across cancers due to TISCH’s annotation biases, with certain subtypes being cancer-specific [28]. Immune cells were identified across most cancer types, with the monocytes/macrophages (Mono/Macro) being annotated in 91% of cancer types. A total of 190 cell-type-specific deconvolved gene expression profiles spanning 17 cell types (including 12 immune cell types) were constructed. Immune infiltration heterogeneity was observed across cancer types (Fig. 2A). To validate TAPE, we generated cell-type-specific expression profiles from independent single-cell RNA-seq data. 76.7% (56/73) of deconvolved matrix pairs from matched cancer-cell type combinations showed significant concordance (Fig. 2B). Literature obtained marker genes for all cell types were overexpressed in the deconvolved gene expression profiles (Fig. 2C). In conclusion, our results highlighted the technical challenges of integrating bulk and single-cell RNA-seq to resolve cell-type-specific expression landscapes.

### Overview of a model for identifying senescent immune cells and samples based on the deconvolution algorithm

**Figure 3:**
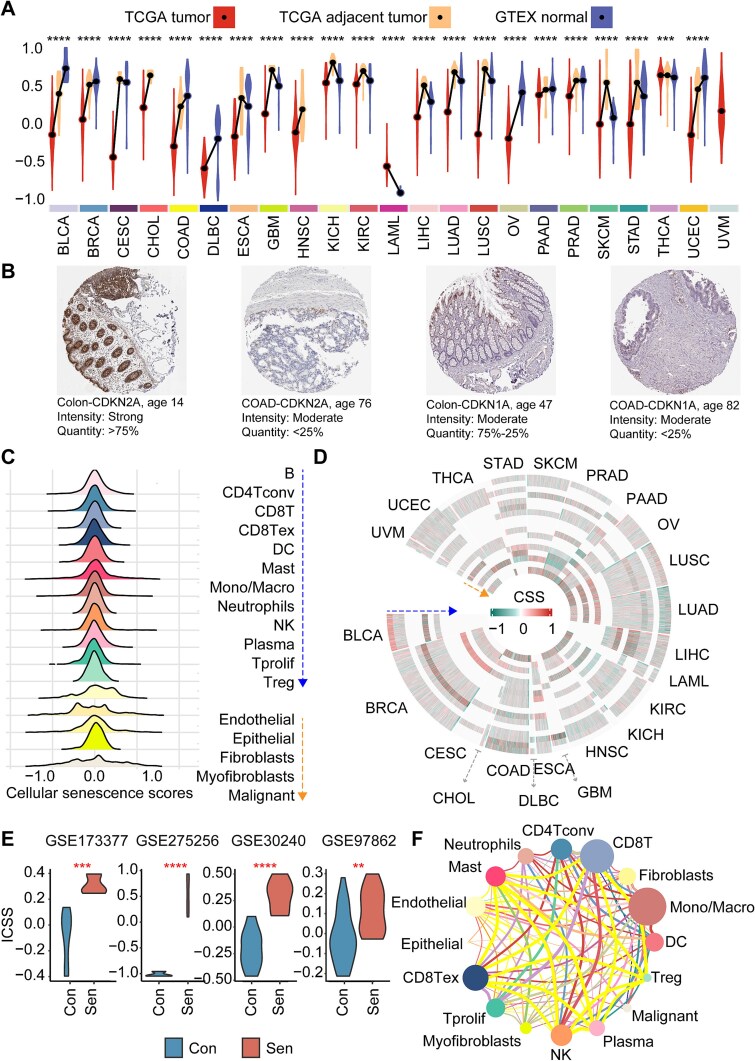
Comprehensive measurement of senescence levels in tissues and cells based on ISENICS across cancer types. (A) Distribution of cellular senescence scores (CSS) in tumor, tumor-adjacent, and normal tissues across different cancers. The points and lines of the violin plot represent the different mean values. (B) IHC staining of CDKN1A (p21) and CDKN2A (p16) in colon tissue and COAD. (C) Ridge diagram illustrates the distribution of cellular senescence scores across cells in various cancers. (D) Circos illustrates the heterogeneity of cellular senescence scores across different cancers and cell types. The sequence of cells from the outer ring to the inner ring was the sequence of cells in the left ridge diagram. (E) Violin plot illustrates comparison of ICSS between different senescence groups. (F) Cell co-senescence network is visualized with node colors indicating cell types, edge colors representing different cancer types involved in co-senescence, edge thickness representing the absolute value of ICSS correlation, and node sizes representing the cumulative thickness of connected edges.

A novel model ISENICS was constructed for quantifying senescent immune cells. We hypothesized that the higher the cellular senescence levels, the greater the difference in activity between senescence-positive and senescence-negative gene sets [12]. The CellAge signature outperformed existing senescence gene sets (SenMayo, siAge, SenCID; Supplementary Fig. 3A), and correlated significantly with SASP gene expression (Supplementary Fig. 3B, C). The ICSS was defined as the difference between the two gene sets of CellAge (Methods). Moreover, Low-ICSS CD8T cells in BRCA exhibited enhanced cytotoxic cytokine production (Supplementary Fig. 3D). Analysis of 8436 tumors, 681 tumor-adjacent tissues, and 5265 normal samples revealed tissue-specific cellular senescence heterogeneity. In most cancers, the cellular senescence scores of normal samples or tumor-adjacent samples was higher than that of tumor samples. The urinary system cancer (KICH, KIRC, PRAD) displayed relatively high cellular senescence scores, whereas hematopoietic cancers (DLBC, LAML) exhibited low cellular senescence scores [4] (Fig. 3A). The pattern in cellular senescence scores distribution suggested that tissue type may play a critical role in influencing the degree of senescence in cancers, and tumor growth potentially represents a mechanism to evade cellular senescence [29, 30]. However, LAML tumors displayed higher senescence levels than normal hematopoietic tissues. LAML cells drive stromal senescence via superoxide production, fostering leukemia-permissive microenvironments [31]. In addition, immunohistochemical staining revealed higher p16/p21 protein expression in young normal colon vs. aged COAD tissues (Fig. 3B). Tumor cells may maintain a lower senescence levels through specific immune escape mechanisms to avoid the immune system’s surveillance and clearance [32].

The cellular senescence scores distribution followed normal distribution, indicating low variance in cellular senescence scores values ( Fig. 3C). The impact of cellular senescence levels on the TIME remains to be fully elucidated across different cancers [ 33]. To gain further insight, we categorized immune cell subtypes into high-, medium-, and low-ICSS groups based on senescence levels ( Fig. 3D). Subsequently, the ICSS differences between senescent and nonsenescent samples were compared. Consistently, ICSS was significantly higher in senescent samples ( Fig. 3E).

Interestingly, in specific cancers, the cell–cell co-senescence was quantified via cellular senescence scores correlation networks in cancer-specific contexts. Mono/Macro cells showed maximal co-senescence connectivity, with the highest cumulative interaction strength. T cells demonstrated pronounced co-senescence with diverse immune populations across pan-cancer. This co-senescence was the most evident in the COAD (Fig. 3F).

### Identifying differentially expressed genes between senescent and nonsenescent immune cells and samples

**Figure 4:**
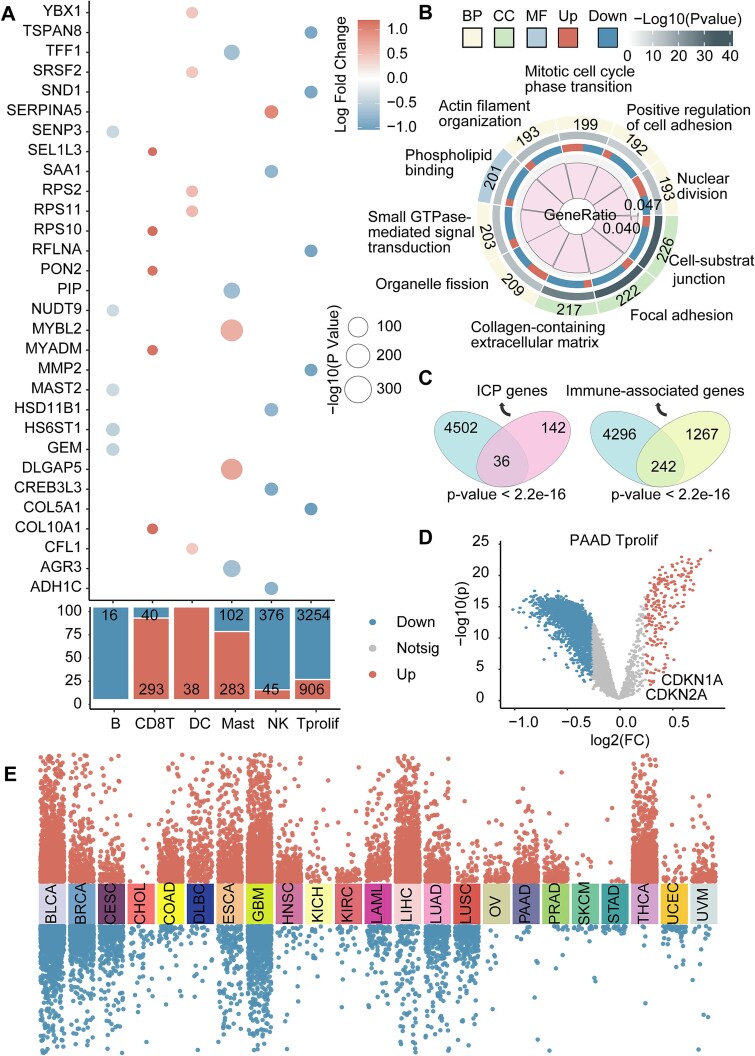
DEGs in senescent immune cells. (A) Dot plot illustrates the top 5 DEGs each cell type between the high- and low-ICSS groups in deconvolved cell-type-specific gene expression profiles. The size of each circle represents the significance levels [−log10 (*P-*values)], while the color of the circle indicates the log2FC. Additionally, the percentage bar chart displaying the proportion and number of up-regulated and down-regulated genes among different immune cell groups. (B) Circus plot illustrates the top 10 enriched reactome pathway terms of immune cell DEGs between the high- and low-ICSS groups and the number of up-regulated and down-regulated genes each pathway contains. (C) Venn diagram illustrates the relationship of DEGs and immune-, and ICP-related genes. The intersection represents the common genes. The hypergeometric test was used to assess the significance of the overlap. (D) A volcano plot showing the log2 fold change of DEGs in PAAD Tprolif cells between ICSS groups. (E) Manhattan plot illustrates the DEGs between the HSTIME and LSTIME samples across various cancers.

To investigate the impact of ICSS heterogeneity in the TIME, we further analyzed deconvolved immune cell expression profiles, revealing DEGs in ICSS groups (Fig. 4A). Pathway enrichment analysis of DEGs between the high- and low-ICSS groups showed significant enrichment in pathways related to immune cell differentiation, cell proliferation, migration, adhesion, and metabolism, all of which might be notably altered by senescence (Fig. 4B). Together, these changes led to a weakened immune cell response to tumors, contributing to the occurrence of chronic low-grade inflammation, also known as “inflammaging” [34]. Understanding these pathway alterations could help uncover the dysfunctions associated with the senescent immune system and provide novel targets for the treatment of senescence-related diseases. Finally, we found that genes associated with senescent immune cells significantly overlapped with genes related to immune function and ICPs [35] (Fig. 4C). Tprolif exhibited the most DEGs. A PAAD-Tprolif volcano plot highlighted ICSS-associated DEGs, including CDKN2A (p16) and CDKN1A (p21) upregulation in high-ICSS group cells (Fig. 4D). For tumor samples, DEGs of HSTIME and LSTIME abundance varied across cancers, with BLCA, GBM, LIHC, and THCA showing the highest numbers (Fig. 4E).

### Exploring TIME and immune signatures based on ISENICS

**Figure 5:**
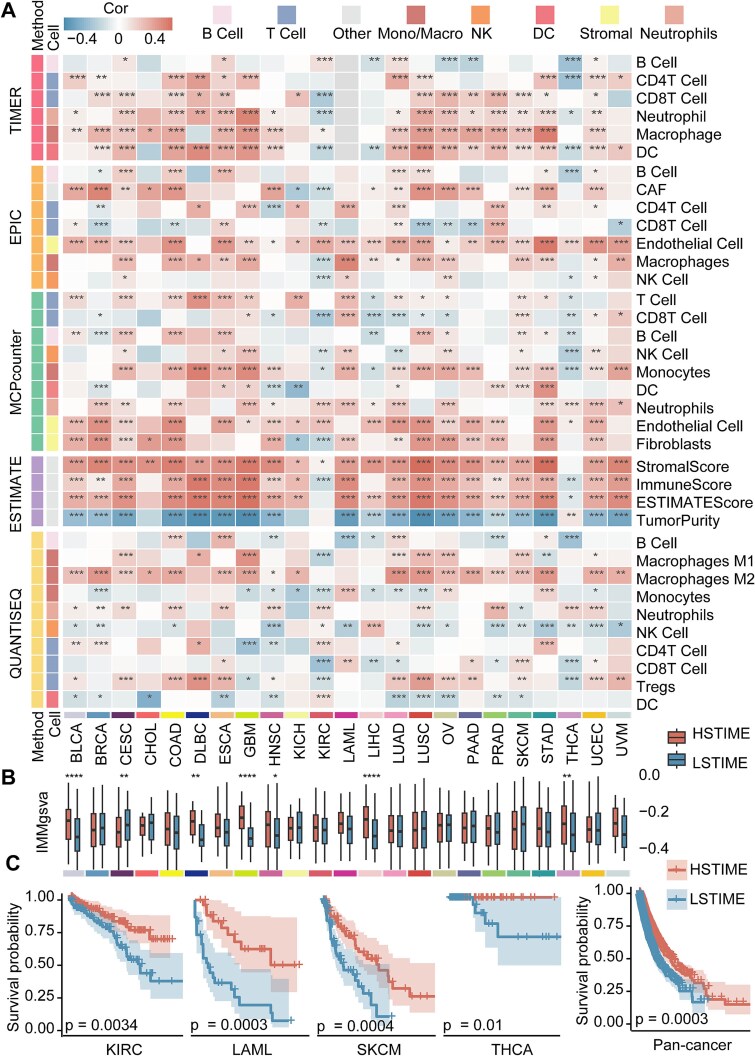
TCGA tumor patients are classified into HSTIME and LSTIME. (A) Heatmap demonstrating the correlation and significance of sample ICSS with the levels of immune infiltration in 23 cancer types. The cancer LAML is not applicable in the TIMER algorithm, so the value is NA. (B) Boxplots illustrates the differences of GSVA scores for immune-related genes (IMMgsva) between the HSTIME and LSTIME patients for individual cancer types. (C) Kaplan–Meier curves of OS in KIRC, LAML, SKCM, THCA, and all 23 cancer samples between the HSTIME and LSTIME patients. The survival difference was calculated by log-rank test. Shaded areas indicate 95% CI.

Senescence levels of immune cells in cancer might lead to a decline in immune function, while this decline recruited more immune cells to generate an immune response to clear the cancer, which was a complex dynamic process [36, 37]. To research the relationship between ICSS and the levels of immune infiltration among tumor samples, we analyzed the infiltration of immune cells using various algorithms [22]. While ICSS was positively associated with immune infiltration in most cancers (18/23), a negative correlation emerged in KIRC and THCA. At the same time, the ESTIMATE data revealed a positive correlation between ICSS and stromal scores, immune scores, and estimate scores, and negative correlation with tumor purity, except in KIRC and THCA (Fig. 5A).

Given the positive relationship between ICSS of tumor samples and immune infiltration, we further explored the combined effect of senescent immune cells in TIME. For example, there were differences in immunity and survival between HSTIME and LSTIME patients. We found that comprehensive immunosenescence resulted in higher immune-related gene scores in HSTIME patients than that in LSTIME patients. This effect was significant in 7 out of 23 cancer types [38] (Fig. 5B). Higher immunosenescence was associated with better prognosis. After FDR correction of *P*-value, the results were as follows: KIRC (*P* = .0034), LAML (*P* = .0003), SKCM (*P* = .0004), and THCA (*P* = .01). To be more meaningfully compare survival outcomes between HSTIME and LSTIME patients, we integrated samples from 23 cancer types, which showed that the HSTIME patients still had a better survival trend than LSTIME patients (*P* = .0003; Fig. 5C). Together, our findings aligned with the theory that tumor samples maintained their malignant phenotype through the senescence escape mechanism.

### Cell interaction variations across ICSS groups

The time required to calculate the cellular senescence scores for GSE115301 using AUCell and GSVA was 2.72 s and 4.97 s, respectively. Thus, it was shown that AUCell speeds up the operation, making it more suitable for large-scale single-cell RNA-seq data. In the GSE115301 dataset, ISENICS (AUC = 0.797) demonstrated slightly higher accuracy than TCSER (AUC = 0.783) (Supplementary Fig. 4).

**Figure 6:**
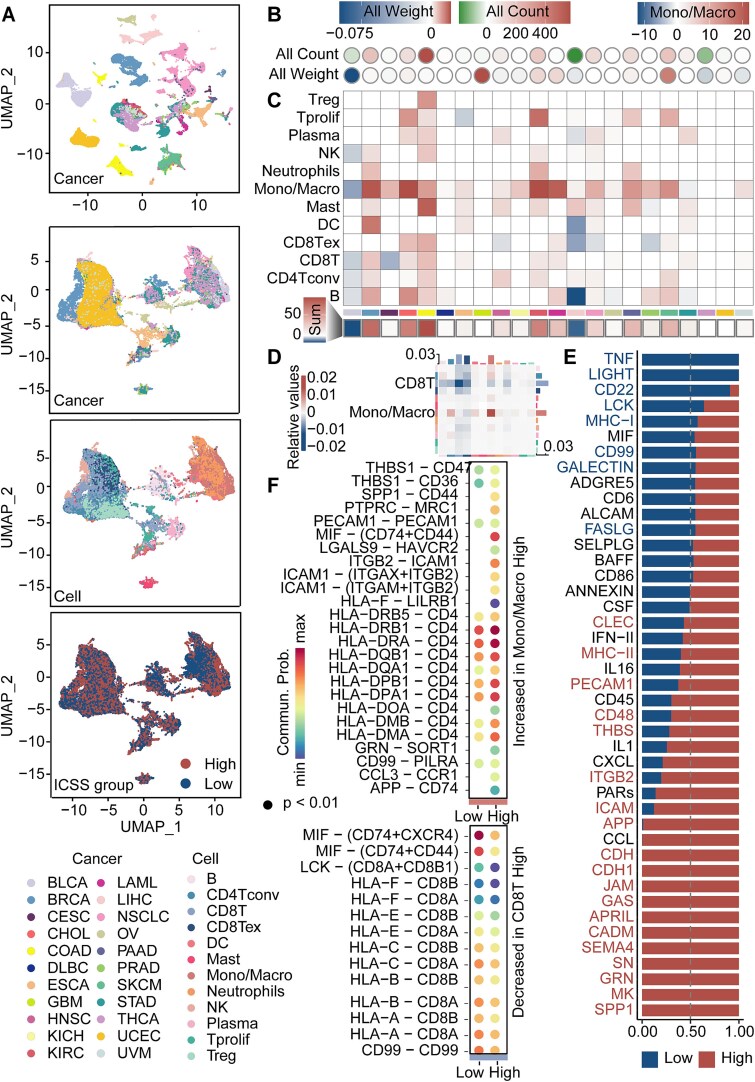
Impact of senescent immune cell on TIME at the single-cell level. (A) UMAP visualization of merged single-cell RNA-seq data of multiple cancers. The major compartment identity of each cancer and cell is shown on the bottom. (B) Circle heatmap comparing the high-ICSS to the low-ICSS group, depicting elevated or reduced cell interaction counts and weights across various cancers. (C) The heatmap representing the change in interaction weight between Mono/Macro cells and other cells, with the total weight change calculated for all interactions within each cancer type. The bottom heatmap is the sum of each column. (D) The heatmap denotes cellular interaction weights. The vertical axis is the cell that sends the signal and the horizontal axis is the cell that receives the signal. (E) Bar plot illustrates differences in signaling pathways among single-cell ICSS groups. (F) Bubble plot illustrates different probabilities of communication of cell-interacting ligand receptor pairs regulation. The size of the dots indicates the *P*-values.

The LUAD and LUSC used a common NSCLC single-cell RNA-seq data for TAPE, so 22 cancer types were included in the single-cell analysis. We presented the distribution of 78 142 cells, excluding medium-ICSS cells, across 12 immune cell types after eliminating the batch effect: B cells, CD4+ conventional T cells (CD4Tconv), CD8+ T cells (CD8T), exhausted CD8+ T cells (CD8Tex), DC, mast cells, monocytes/macrophages (Mono/Macro), neutrophils, NK cells, plasma cells, Tprolif, and Treg cells (Fig. 6A).

The overall count and weight of cell interactions in the high-ICSS group were higher than in the low-ICSS group across most cancer types [39, 40] (Fig. 6B). Taking the Mono/Macro cell population as an example, which had the highest cell count, we found that cell interaction weight varied across cancers when these cells underwent senescence. The self-communication of Mono/Macro was the most pronounced change in the weight of intercell communication. Specifically, the sum interaction weight of immune cells increased most significantly in COAD, whereas it decreased most significantly in BLCA (Fig. 6C).

In the communication analysis of integrated immune single-cell RNA-seq data for pan-cancer, statistical analysis was performed on the weight of intercell communication. Mono/Macro and CD8T cells with the largest differences in cell interactions between ICSS groups were paid special attention. Compared to low-ICSS, high-ICSS had obviously more Mono/Macro interact signals and obviously lower CD8T interact signals (Fig. 6D). We analyzed the conserved and specific signaling pathways and found that TNF [41] and LIGHT [42] were signaling pathways unique to the low-ICSS group of immune cells (Fig. 6E). After immune cell senescence, the ligand-receptor interactions might undergo upregulation between Mono/Macro ICSS groups or downregulation between CD8T ICSS groups (Fig. 6F). These results were crucial for understanding how senescent immune cells respond to signals and modulate immune response.

### Enrichment of senescent immune cells was significantly associated with worse immunotherapy response

**Figure 7:**
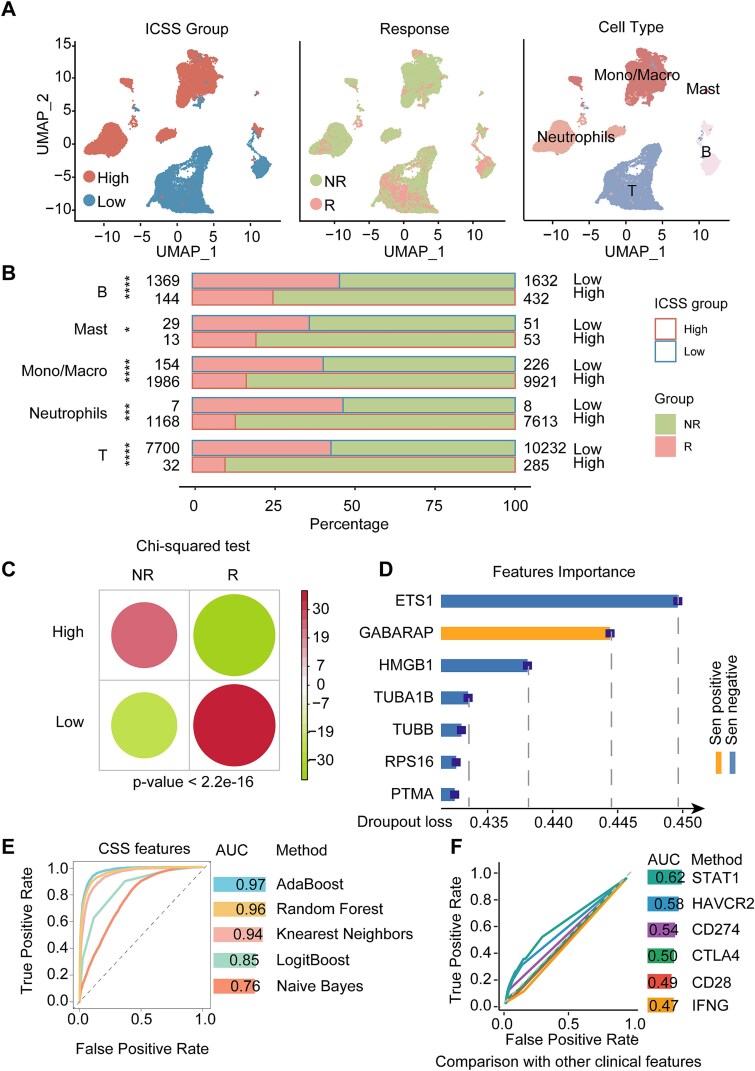
Relationships between senescent immune cells and response to immunotherapy. (A) Cells were clustered into subtypes to draw UMAP. (B) Percentage bars illustrate the proportion of senescent cells from immune responders or nonresponders. (C) Heatmap illustrates the correlation of ICSS groups and immune response groups of all scRNA-seq data. The chi-square test was calculated. (D) Assessment of the importance of genes to our predictor. (E) Five methods were used to plot the receiver operating characteristic (ROC) curves and calculated AUC values of the immune response classifier. (F) ROC curves and AUC values for classifiers of other clinical features associated with immune response.

Senescence levels of immune cells are potential predictors of immunotherapy response [43]. To explore the proportion of immune cells generating an immunotherapy response between ICSS groups, we calculated the ICSS of all immune cells from immunotherapy response single-cell RNA-seq data (Fig. 7A) and found that the high-ICSS cells were worse at immunotherapy response (Fig. 7B and C).

In order to construct the immune response prediction model, we further screened key genes. Firstly, the DEGs were calculated for the ICSS groups of immune cells. Secondly, 62 significant genes were obtained by univariate Cox regression analysis and LASSO regression on the immunotherapy response. In addition, genes from the intersection of 62 model genes and CellAge genes were identified for importance evaluation (Fig. 7D). The key predictive factors in the model had multiple effects. For example, ETS1 can regulate ribosomal protein genes, delay cell senescence, and relate to the expression of multiple ICP molecules [44]. We used these 62 genes to predict immunotherapy response based on some machine learning algorithms (Fig. 7E). Compared to other immunotherapy response-related clinical features, our model had a higher AUC than STAT1, HAVCR2, CD274, CTLA4, IFNG, and CD28 (Fig. 7F).

## Discussion

This study presented a novel model, ISENICS, for quantifying senescent immune cell characteristics in the TIME and exploring their clinical implications. The work leveraged bulk RNA-seq, single-cell RNA-seq, and clinical data from TCGA, GTEx, GEO, and TISCH, covering 23 cancer types and over 14 000 samples, and combined the TAPE deep learning deconvolution algorithm with ICSS, offering a new tool for studying senescent immune cells. This versatility allows us to leverage a limited amount of data to obtain more information about cell heterogeneity, reducing the cost and time of research.

There were some advantages and biological interpretability of the ISENICS: (i) ISENICS evaluated senescence levels of single immune cells by deconvolution, and revealed the heterogeneity of senescent diverse immune cell types across cancers. (ii) ISENICS could help explore the roles of senescent immune cells in TIME, such as interactions between senescent immune cells and other cell types. (iii) In contrast to previous analyses describing cellular senescence at the level of the whole tumor, ISENICS divided tumor patients into the high and low senescent TIME groups (HSTIME and LSTIME), which helped explore the roles of senescent immune cells in TIME, such as patient survival and immune infiltration. (iv) ISENICS revealed the inhibitory effect of immunosenescence on immunotherapy response.

There were still some limitations when constructing the ISENICS. Although we analyzed the existing senescence-related gene sets and selected the most accurate gene set (CellAge) to apply to our model, ISENICS, the selection of different gene sets still affects the efficiency of the whole model. Another challenge was the validation of ICSS, which was primarily constructed based on computationally derived gene expression patterns. Therefore, experimental verification will be necessary in future studies. In addition, we found only one single-cell RNA-seq data labeled with the senescent status for demonstrating that ISENICS is slightly more accurate than TCSER. To further validate the robustness of ISENICS, more datasets and deconvolution methods should be explored in future studies. In summary, our multiparametric evaluation criteria revealed common features and heterogeneity of senescent immune cells. ISENICS-based assessment offers a novel approach to understand the mechanisms of TIME at a systemic level with a scalable approach. This model not only aids in characterizing the roles of senescent immune cells but also provides insights into the complex interactions within the TIME.

**Key Points**

- The model Identifying Senescent Immune Cells and Samples (ISENICS) was developed across 23 cancer types by integrating bulk and single-cell RNA-seq data.
- ISENICS could categorize tumor patients into subgroups with different levels of immune cellular senescence scores (ICSS).
- The overall count and weight of cell interactions in the high-ICSS group were higher than those in the low-ICSS group across most cancer types.
- Immune cellular senescence status not only effectively predicts better survival prognosis, but also strongly correlates with poorer immunotherapy response.

## Supplementary Material

Supplemental_file_bbaf469

## Data Availability

The data supporting this study’s findings are available from the UCSC Xena platform (http://xena.ucsc.edu/). Immunotherapy response and other single-cell RNA-seq data were obtained from the GEO database. The analysis results associated with this paper are available on GitHub (https://github.com/TianMiaoMiaoWorkspace/ISENICS/).
