# Supplementary material for: ISENICS: a model for identifying senescent immune cells and samples and characterization of their roles in tumor microenvironment
Source: Brief Bioinform. 2025 Sep 11;26(5):bbaf469. doi: 10.1093/bib/bbaf469 (PMC12423394; doi:10.1093/bib/bbaf469)
Supplement: Supplemental_file_bbaf469 [file supplemental_file_bbaf469.docx]

**Supplementary Table 1.** TCGA dataset sources and sample size statistics. The three columns of the table correspond to the cancer abbreviation in TCGA, sample size, and cancer details, respectively.

| **TCGA** | **Num** | **Detail** |
| --- | --- | --- |
| BLCA | 414 | Bladder Urothelial Carcinoma |
| BRCA | 1109 | Breast invasive carcinoma |
| CESC | 306 | Cervical squamous cell carcinoma and endocervical adenocarcinoma |
| CHOL | 36 | Cholangiocarcinoma |
| COAD | 480 | Colon adenocarcinoma |
| DLBC | 48 | Lymphoid Neoplasm Diffuse Large B-cell Lymphoma |
| ESCA | 162 | Esophageal carcinoma |
| GBM | 169 | Glioblastoma multiforme |
| HNSC | 502 | Head and Neck squamous cell carcinoma |
| KICH | 65 | Kidney Chromophobe |
| KIRC | 539 | Kidney renal clear cell carcinoma |
| LAML | 151 | Acute Myeloid Leukemia |
| LIHC | 374 | Liver hepatocellular carcinoma |
| LUAD | 535 | Lung adenocarcinoma |
| LUSC | 502 | Lung squamous cell carcinoma |
| OV | 379 | Ovarian serous cystadenocarcinoma |
| PAAD | 178 | Pancreatic adenocarcinoma |
| PRAD | 499 | Prostate adenocarcinoma |
| SKCM | 471 | Skin Cutaneous Melanoma |
| STAD | 375 | Stomach adenocarcinoma |
| THCA | 510 | Thyroid carcinoma |
| UCEC | 552 | Uterine Corpus Endometrial Carcinoma |
| UVM | 80 | Uveal Melanoma |

**Supplementary Table 2.** Detailed information of 80 immune cell samples, including sample sources, corresponding immune cell subtypes and cellular senescence scores for evaluating ICSS predictive efficacy.

| **GEO** | **Sample** | **Cell type** | **SenMayo** | **SenCID** | **siAge** | **CellAge** | **Group** |
| --- | --- | --- | --- | --- | --- | --- | --- |
| GSE30240 | GSM748845 | TK6_cells | 0.04 | 0.11 | 0.18 | 0.1 | con |
| GSE30240 | GSM748846 | TK6_cells | -0.26 | 0.12 | 0.18 | -0.29 | con |
| GSE30240 | GSM748847 | TK6_cells | 0.07 | 0.02 | 0.07 | -0.3 | con |
| GSE30240 | GSM748848 | TK6_cells | 0.08 | 0.1 | 0.45 | -0.04 | con |
| GSE30240 | GSM748849 | TK6_cells | -0.03 | -0.21 | -0.38 | -0.1 | con |
| GSE30240 | GSM748850 | TK6_cells | -0.14 | 0.28 | 0.15 | -0.39 | con |
| GSE30240 | GSM748851 | TK6_cells | 0.01 | 0.03 | -0.1 | -0.11 | con |
| GSE30240 | GSM748852 | TK6_cells | 0 | -0.19 | -0.12 | -0.22 | con |
| GSE30240 | GSM748853 | TK6_cells | 0.06 | 0.06 | -0.09 | -0.46 | con |
| GSE30240 | GSM748854 | TK6_cells | 0.19 | -0.04 | 0.32 | 0.29 | sen |
| GSE30240 | GSM748855 | TK6_cells | -0.11 | -0.08 | -0.13 | 0.11 | sen |
| GSE30240 | GSM748856 | TK6_cells | -0.01 | -0.12 | 0.05 | 0.13 | sen |
| GSE30240 | GSM748857 | TK6_cells | 0.11 | 0.07 | 0.28 | 0.5 | sen |
| GSE30240 | GSM748858 | TK6_cells | 0.03 | 0.02 | -0.31 | 0.36 | sen |
| GSE30240 | GSM748859 | TK6_cells | 0.13 | -0.2 | -0.05 | 0.38 | sen |
| GSE173377 | SMB822 | T_cells | 0.06 | -0.03 | 0.38 | 0.4 | sen |
| GSE173377 | SMB1283 | T_cells | 0.04 | -0.05 | 0.22 | 0.25 | sen |
| GSE173377 | SMB1285 | T_cells | 0.1 | -0.07 | 0.65 | 0.29 | sen |
| GSE173377 | SMB821 | T_cells | -0.29 | -0.11 | -0.46 | 0.04 | con |
| GSE173377 | SMB1267 | T_cells | 0.19 | -0.21 | -0.58 | -0.04 | con |
| GSE173377 | SMB1269 | T_cells | -0.01 | -0.09 | -0.75 | 0.02 | con |
| GSE173377 | SMB825 | T_cells | -0.07 | -0.08 | 0.27 | 0.11 | con |
| GSE173377 | SMB1271 | T_cells | -0.08 | 0.06 | -0.23 | -0.38 | con |
| GSE173377 | SMB1273 | T_cells | 0.02 | 0.08 | -0.57 | -0.4 | con |
| GSE173377 | SMB826 | T_cells | 0.17 | 0.02 | 0.08 | -0.01 | con |
| GSE173377 | SMB1275 | T_cells | 0.15 | 0 | 0.47 | -0.19 | con |
| GSE173377 | SMB1277 | T_cells | 0.24 | -0.15 | 0.53 | 0.14 | con |
| GSE275256 | SLO_1-2_1 | Microglia_like_cells | 0.29 | -0.14 | 0.2 | 0.39 | sen |
| GSE275256 | SLO_1-2_2 | Microglia_like_cells | 0.32 | -0.32 | 0.26 | 0.1 | sen |
| GSE275256 | SLO_1-2_3 | Microglia_like_cells | 0.34 | -0.08 | 0.03 | 0.28 | sen |
| GSE275256 | SLO_1_1 | Microglia_like_cells | 0.21 | -0.09 | -0.21 | 0.89 | sen |
| GSE275256 | SLO_1_2 | Microglia_like_cells | 0.07 | -0.38 | -0.24 | 0.89 | sen |
| GSE275256 | SLO_1_3 | Microglia_like_cells | 0.17 | -0.2 | -0.06 | 0.93 | sen |
| GSE275256 | VEH_1 | Microglia_like_cells | -0.46 | 0.14 | 0.1 | -0.96 | con |
| GSE275256 | VEH_2 | Microglia_like_cells | -0.47 | 0.1 | 0.17 | -1.05 | con |
| GSE275256 | VEH_3 | Microglia_like_cells | -0.47 | 0.18 | -0.15 | -1.03 | con |
| GSE97862 | TU0053_naive_RNASeq | CD8T_cells | -0.39 | -0.04 | -1.09 | -0.1 | con |
| GSE97862 | TU0053_TCM_RNASeq | CD8T_cells | -0.16 | 0.17 | 0.18 | -0.2 | con |
| GSE97862 | TU0053_TEM_RNASeq | CD8T_cells | 0.02 | 0.17 | 0.4 | -0.15 | con |
| GSE97862 | TU0053_TEMRA_RNASeq | CD8T_cells | -0.27 | 0.12 | 0.68 | 0.05 | sen |
| GSE97862 | TU0054_naive_RNASeq | CD8T_cells | -0.4 | -0.07 | -0.92 | -0.16 | con |
| GSE97862 | TU0054_TCM_RNASeq | CD8T_cells | -0.32 | 0.11 | 0.02 | -0.21 | con |
| GSE97862 | TU0054_TEM_RNASeq | CD8T_cells | -0.08 | 0.14 | 0.45 | -0.21 | con |
| GSE97862 | TU0054_TEMRA_RNASeq | CD8T_cells | -0.28 | 0.11 | 0.32 | 0.04 | sen |
| GSE97862 | TU0057_naive_RNASeq | CD8T_cells | -0.26 | -0.17 | -0.92 | 0.02 | con |
| GSE97862 | TU0057_TCM_RNASeq | CD8T_cells | -0.2 | 0.08 | 0.03 | -0.07 | con |
| GSE97862 | TU0057_TEM_RNASeq | CD8T_cells | 0.08 | 0.16 | 0.25 | 0.01 | con |
| GSE97862 | TU0057_TEMRA_RNASeq | CD8T_cells | -0.19 | 0.04 | 0.17 | 0 | sen |
| GSE97862 | TU0079_naive_RNASeq | CD8T_cells | -0.34 | -0.1 | -1.06 | -0.04 | con |
| GSE97862 | TU0079_TCM_RNASeq | CD8T_cells | -0.28 | 0.16 | -0.1 | -0.1 | con |
| GSE97862 | TU0079_TEM_RNASeq | CD8T_cells | -0.27 | 0.02 | 0.4 | 0.01 | con |
| GSE97862 | TU0079_TEMRA_RNASeq | CD8T_cells | -0.26 | 0.19 | 0.2 | -0.03 | sen |
| GSE97862 | TU0084_naive_RNASeq | CD8T_cells | -0.41 | -0.16 | -1.08 | -0.14 | con |
| GSE97862 | TU0084_TCM_RNASeq | CD8T_cells | -0.3 | 0.01 | -0.32 | -0.12 | con |
| GSE97862 | TU0084_TEM_RNASeq | CD8T_cells | -0.23 | 0.01 | 0.17 | -0.06 | con |
| GSE97862 | TU0084_TEMRA_RNASeq | CD8T_cells | -0.08 | -0.09 | 0.18 | 0.14 | sen |
| GSE97862 | TU0085_naive_RNASeq | CD8T_cells | -0.23 | -0.06 | -1.03 | -0.21 | con |
| GSE97862 | TU0085_TCM_RNASeq | CD8T_cells | -0.38 | -0.07 | -0.2 | -0.1 | con |
| GSE97862 | TU0085_TEM_RNASeq | CD8T_cells | 0 | -0.14 | 0.43 | -0.04 | con |
| GSE97862 | TU0085_TEMRA_RNASeq | CD8T_cells | 0.09 | -0.14 | 0.41 | 0 | sen |
| GSE97862 | GS0089_naive_RNASeq | CD8T_cells | 0.01 | -0.28 | -0.65 | 0.19 | con |
| GSE97862 | GS0089_TCM_RNASeq | CD8T_cells | 0.24 | -0.03 | 0.02 | 0.01 | con |
| GSE97862 | GS0089_TEM_RNASeq | CD8T_cells | 0.33 | -0.05 | 0.63 | 0.13 | con |
| GSE97862 | GS0089_TEMRA_RNASeq | CD8T_cells | 0.33 | -0.02 | 0.02 | 0.21 | sen |
| GSE97862 | GS0098_naive_RNASeq | CD8T_cells | 0.1 | -0.13 | -0.93 | 0.17 | con |
| GSE97862 | GS0098_TCM_RNASeq | CD8T_cells | 0.32 | -0.01 | 0.13 | 0.19 | con |
| GSE97862 | GS0098_TEM_RNASeq | CD8T_cells | 0.38 | 0.01 | 0.19 | 0.11 | con |
| GSE97862 | GS0098_TEMRA_RNASeq | CD8T_cells | 0.41 | -0.08 | 0.41 | 0.3 | sen |
| GSE97862 | GS0108_naive_RNASeq | CD8T_cells | 0.16 | -0.26 | -0.54 | 0.09 | con |
| GSE97862 | GS0108_TCM_RNASeq | CD8T_cells | 0.19 | -0.06 | 0.15 | -0.07 | con |
| GSE97862 | GS0108_TEM_RNASeq | CD8T_cells | 0.36 | 0.07 | 0.45 | -0.01 | con |
| GSE97862 | GS0108_TEMRA_RNASeq | CD8T_cells | 0.45 | 0.02 | 0.43 | 0.2 | sen |
| GSE97862 | GS0117_naive_RNASeq | CD8T_cells | 0.12 | -0.28 | -0.46 | 0.28 | con |
| GSE97862 | GS0117_TCM_RNASeq | CD8T_cells | 0.24 | -0.05 | 0.07 | 0 | con |
| GSE97862 | GS0117_TEM_RNASeq | CD8T_cells | 0.33 | 0.05 | 0.73 | 0.08 | con |
| GSE97862 | GS0117_TEMRA_RNASeq | CD8T_cells | 0.19 | -0.1 | 0.99 | 0.24 | sen |
| GSE97862 | GS0259_naive_RNASeq | CD8T_cells | 0.16 | -0.12 | -0.59 | -0.01 | con |
| GSE97862 | GS0259_TCM_RNASeq | CD8T_cells | 0.35 | -0.02 | 0.09 | 0.15 | con |
| GSE97862 | GS0259_TEM_RNASeq | CD8T_cells | 0.45 | 0.13 | 0.88 | -0.07 | con |
| GSE97862 | GS0259_TEMRA_RNASeq | CD8T_cells | 0.37 | -0.07 | 0.92 | 0.13 | sen |


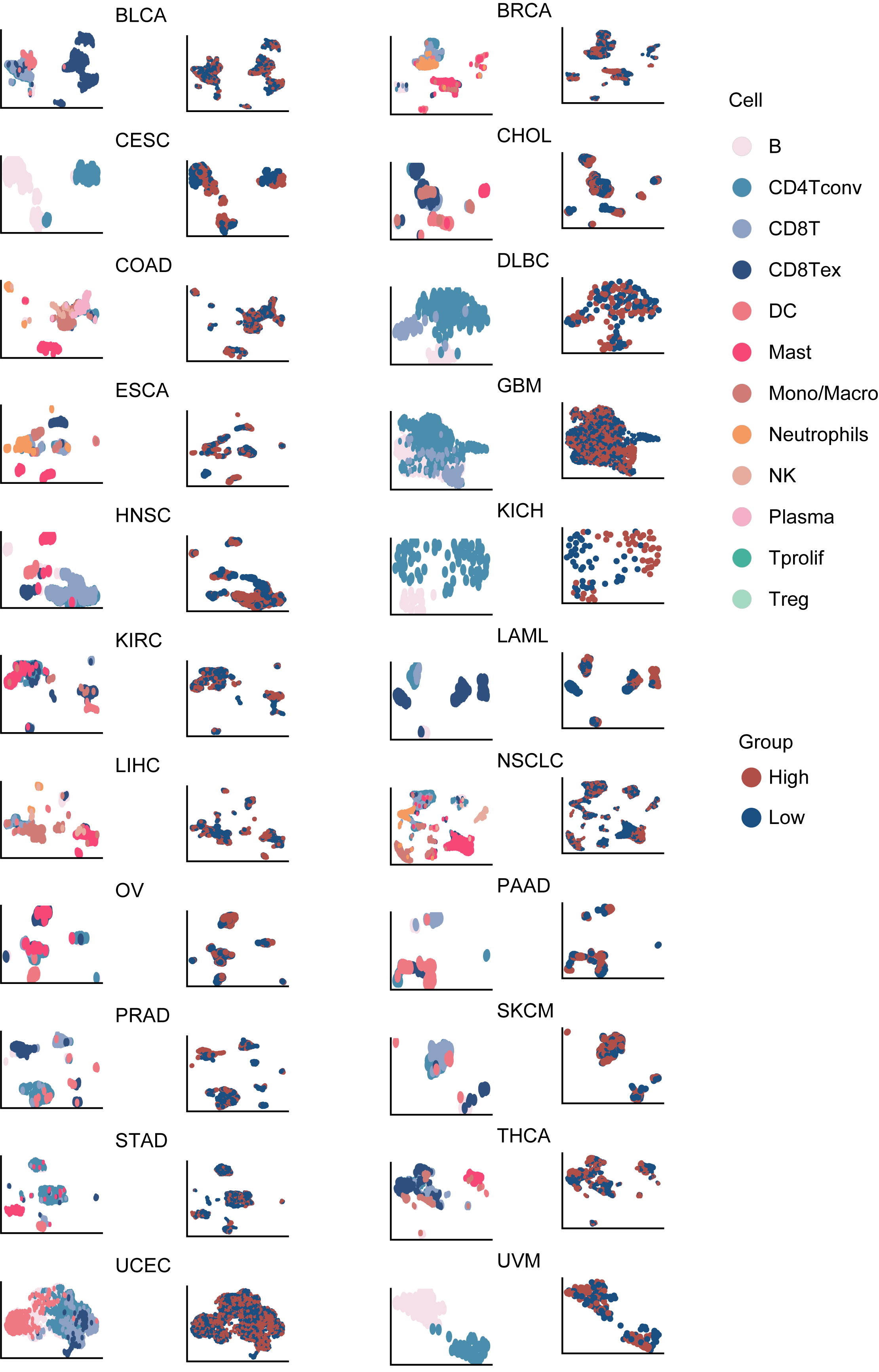


**Supplementary Figure 1.** Single-cell UMAP comparison of ICSS in different immune cell subtypes.

**Alt text :** UMAP of ICSS across immune cell subtypes.


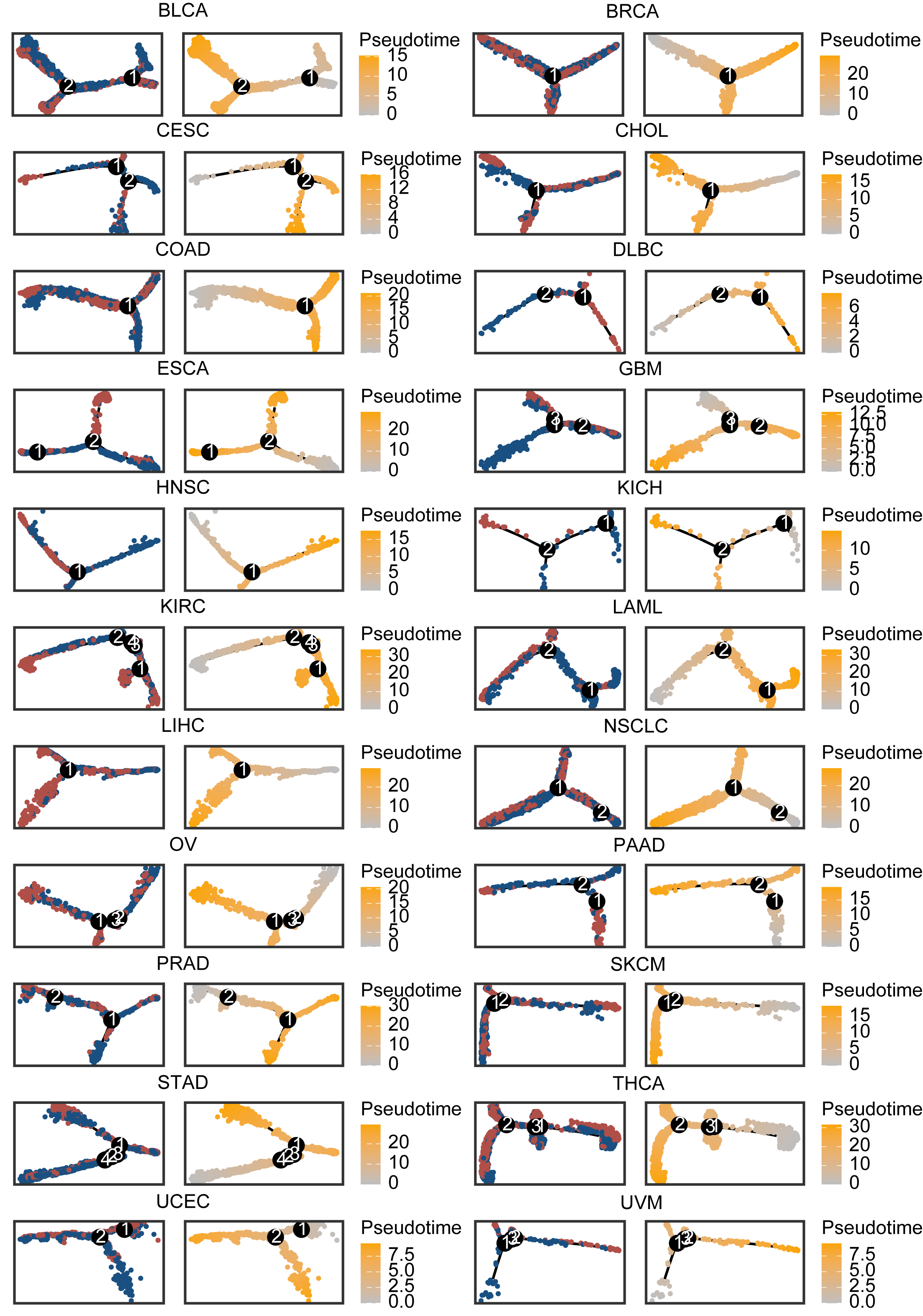


**Supplementary Figure 2.** Single-cell proposed time-series analysis demonstrates changes in immune cellular senescence over time in different cancer subtypes.

**Alt text :** Time-series analysis of senescent immune cells.

**
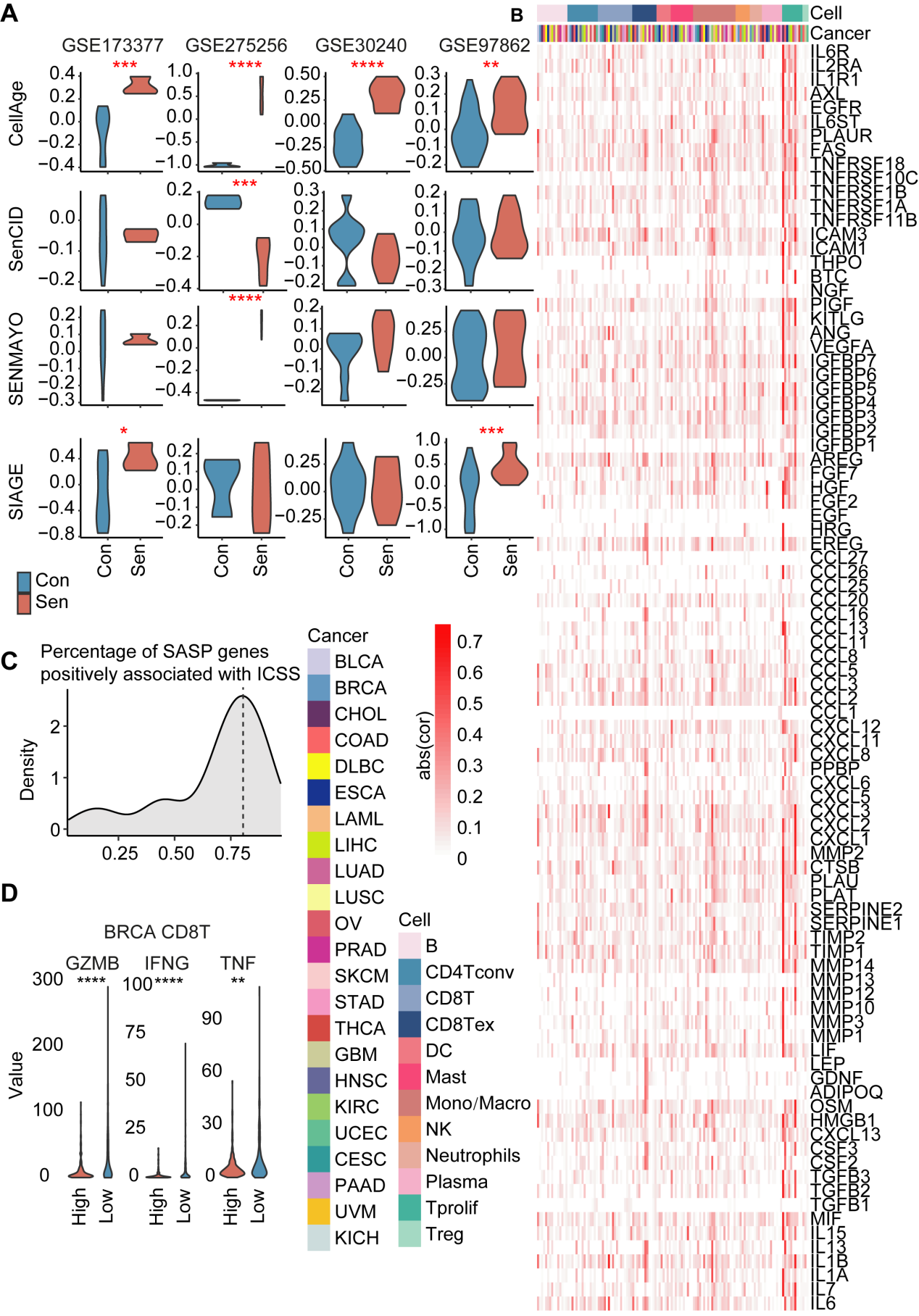
**

**Supplementary Figure 3.** ICSS accuracy verification. (**A**) Immune cellular senescence calculated by CellAge, siAge, SenMayo, and SenCID gene sets. (**B**) The absolute value of the correlation between ICSS and SASP genes in each deconvolved expression profile. (**C**) Density map of the proportion of SASP genes positive associated with ICSS. (**D**) Violin plots illustrate the different expression of cytotoxic cytokines in CD8T ICSS groups of BRCA.

**Alt text :** The CellAge gene set was selected for ISENICS and its accuracy in quantifying immune cellular senescence levels was validated.


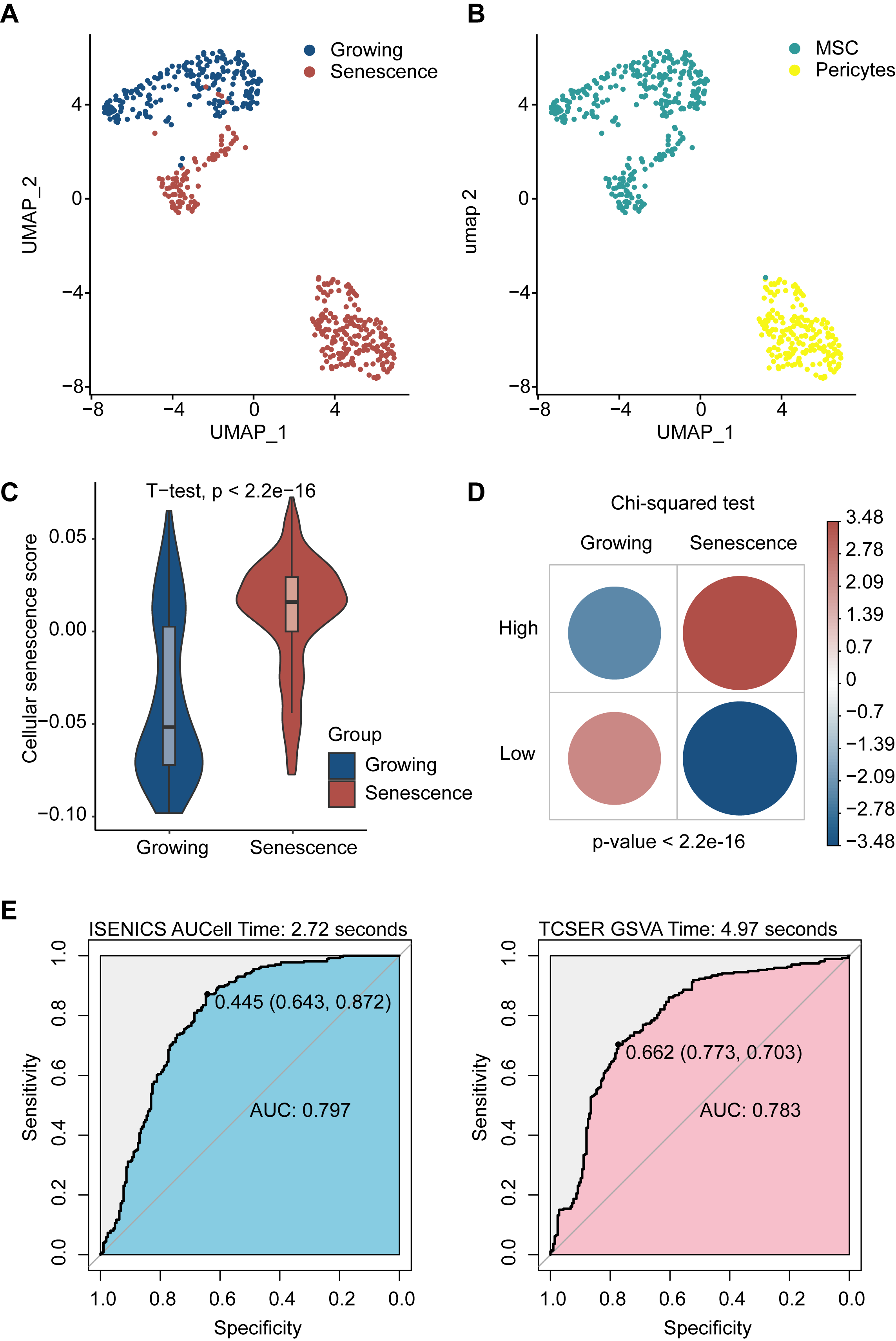


**Supplementary Figure 4.** Cellular senescence accuracy verification in single-cell RNA-seq data. (**A**) UMAP plot of GSE115301 dataset with cellular senescence labels. (**B**) The cells of GSE115301 were clustered into two cell subtypes: mesenchymal stem cells (MSCs) and pericytes. (**C**) The violin plot indicated that the cellular senescence scores of senescent cells were significantly higher than those of growing cells. (**D**) The chi-square test compared the senescence status of MSCs with the cellular senescence groups and showed a significant correlation. (**E**) The ROC curve showed the comparison of the accuracy and efficiency between the AUCell algorithm of ISENICS and the GSVA algorithm of TCSER for single-cell RNA-seq data was performed.

**Alt text :** ISENICS accuracy was verified in GSE115301 and compared with that of the TCSER algorithms.
